# Dynamics of probing a quantum-dot spin qubit with superconducting resonator photons

**DOI:** 10.1038/s41598-018-34108-0

**Published:** 2018-10-25

**Authors:** Xing-Yu Zhu, Tao Tu, Ao-Lin Guo, Zong-quan Zhou, Chuan-Feng Li, Guang-Can Guo

**Affiliations:** 1 Key Lab of Quantum Information, Chinese Academy of Sciences, University of Science and Technology of China, Hefei, 230026 China; 2 Department of Physics and Astronomy, University of California at Los Angeles, California, 90095 USA

## Abstract

The hybrid system of electron spins and resonator photons is an attractive architecture for quantum computing owing to the long coherence times of spins and the promise of long-distance coupling between arbitrary pairs of qubits via photons. For the device to serve as a building block for a quantum processer, it is also necessary to readout the spin qubit state. Here we analyze in detail the measurement process of an electron spin singlet-triplet qubit in quantum dots using a coupled superconducting resonator. We show that the states of the spin singlet-triplet qubit lead to readily observable features in the spectrum of a microwave field through the resonator. These features provide useful information on the hybrid system. Moreover, we discuss the working points which can be implemented with high performance in the current state-of-the-art devices. These results can be used to construct the high fidelity measurement toolbox in the spin-circuit QED system.

## Introduction

The ability to couple and manipulate qubit with the help of well-controlled electromagnetic fields has played an important role in the field of cavity quantum electrodynamics (CQED)^1–3^. For solid-state circuits, superconducting transmission line resonator has led to significant advances in quantum information processing^4,5^. Prominent examples range from using a resonator quantum bus to generate entanglement between spatially separated qubits^6–8^, utilizing the large Hilbert space of the resonator for a quantum memory^9–11^, and making use of the resonator for quantum non-demolition (QND) measurements^12–15^.

Spin qubits in semiconductor quantum dots are one of the leading candidates for scalable quantum computation^16,17^. There are various encodings of spin qubits into one, two- and three-spin subspaces of electrons in quantum dots^18^. Initialization, single- and two-qubit gate operations, and measurement are the fundamental elements for universal quantum computation. In general, they are required to be fast and with high fidelity to reach the fault-tolerance thresholds. In the past years, several experiments demonstrated a high single-qubit gate fidelity above 99%^19–21^ and a two-qubit gate which can be improved further^22–25^. However, readout of a spin-qubit has received fewer studies than control, which is detrimental for implementing measurement-based protocols such as error-correcting codes^26,27^. In a conventional quantum dot experiment, the readout is slow since it relies on spin-selective tunneling to a lead^28,29^. In contrast, in a typical CQED architecture, the resonator photon has the virtue of fast readout of the qubit with high fidelity^4,5,12^. Here we present a hybrid system of spin singlet-triplet qubit^30^ and resonator photons which benefits from the different advantages of these two distinct elements. In particular, our approach provides an alternative to standard measurement technique for quantum dots, which will be useful to settle remaining key challenges with building spin-based quantum processors.

**Figure 1:**
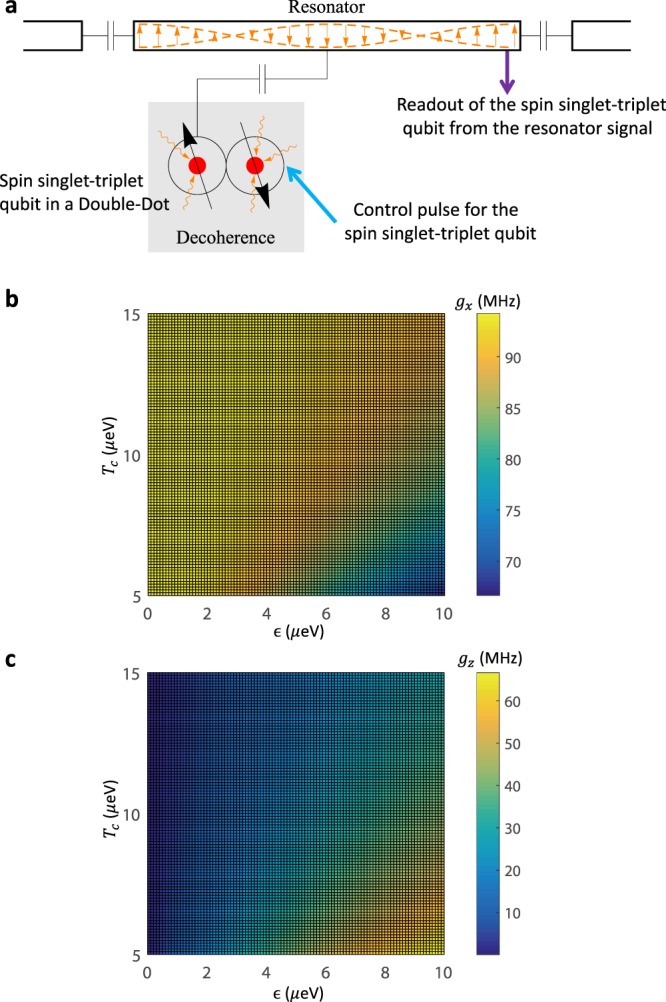
(**a**) Schematic of two-electron spin single-triplet qubit in double quantum dots coupled to photons in a transmission line resonator. The control pulse for the spin qubit can be applied through the gate voltage of the quantum dot. In realistic setup, the spin qubit is influenced by the decoherence effects from the environment noises. The state of the spin qubit can be effectively determined by measuring the signal of a microwave field transmitted through the resonator. (**b**) For this spin-photon coupled system, the effective transverse coupling *g*_*x*_ and longitudinal coupling *g*_*z*_ are plotted as functions of the gate voltage $\epsilon$ and inter-dot tunneling *T*_*c*_.

Our basic scheme involves the double quantum dot and the superconducting transmission line resonator patterned onto a semiconductor substrate such as GaAs, for a schematic illustration, see Fig. 1a. Because of the intrinsic charge property of the spin singlet-triplet states, the resonator generates a well-controlled electric potential landscape for electrons confined in double quantum dot. We show that the electron’s potential can be effectively described by a gate tunable coupling between spin singlet-triplet qubit and resonator photon. Based on an equation of motion approach, then we analyze in detail the time-dependent response of the resonator photon to changes in the qubit state. Intuitively, such results be understood from the fact that the spin singlet state and triplet state have different susceptibilities, therefore we can observe a difference in the resonator signal for the singlet when compared to the triplet. We note that in a previous work^31^, the authors showed the dispersive readout of single-electron spin qubit state with the superconducting resonator signal, which is a first step toward the goal of using the resonator to realize the high performance measurement. Here we go further by identifying the relevant figure of merit for this probing process and showing how the system parameters and corresponding performance can be engineered and tuned. Consequently, we identify strategies to meet these realistic parameters with state-of-the-art experimental techniques and find optimal working regimes. Thanks to the generic nature of our analysis and the variety of system parameters, our framework is readily applicable to a broad class of quantum dot circuits for quantum information platforms.

## Results

### Hamiltonian of the hybrid system with electron spin in quantum dot and photon in resonator

It is hard to couple a single electron spin with a photon in the resonator since the magnetic dipole momentum of an electron spin is rather small, where the direct spin-photon coupling strength is estimated of 10 Hz^32^. Alternatively, the interaction between the charge degrees of freedom of electrons and the electric field of the resonator is very strong. Then the electron spin states can have particular charge character through the spin-charge hybrid method. Therefore this gives rise to a large effective spin-photon coupling strength of MHz. Recently, CQED architectures have been proposed and implemented to couple various electron spin qubits in quantum dots with a superconducting resonator^30,33–43^. These initial demonstrations motivate our research for probing the spin qubit using resonator photons that bring the generality and flexibility of CQED to the electron spin setting.

As shown in Fig. 1a, the CQED setup consists of two elements: two-electron spin qubit in a GaAs double quantum dot, and a superconducting transmission line resonator. The two-electron spin singlet-triplet qubit is defined in the subspace of $|(1,1){T}_{0}\rangle =(|\uparrow \downarrow \rangle -|\downarrow \uparrow \rangle )/\sqrt{2}$, and $|(1,1)S\rangle =(|\uparrow \downarrow \rangle +|\downarrow \uparrow \rangle )/\sqrt{2}$. Here (*n*_*L*_, *n*_*R*_) denotes the number of electrons in the left and right dot, ↑ and ↓ labels spin up and down. The other two spin triplet states, $|(1,1){T}_{+}\rangle =|\uparrow \uparrow \rangle$ and $|(1,1){T}_{-}\rangle =|\downarrow \downarrow \rangle$ are split by an external magnetic field and can be neglected in the following. In the realistic situation^30,44^, we also include the third level: the singlet state with two electrons in the right dot $|(0,2)S\rangle$. Thus, this quantum dot system can be described by a Hamiltonian for $|(1,1){T}_{0}\rangle$, $|(1,1)S\rangle$ and $|(0,2)S\rangle$:

1 $${H}_{QD}=(\begin{array}{ccc}0 & {\rm{\Delta }}B & 0\\ {\rm{\Delta }}B & 0 & {T}_{c}\\ 0 & {T}_{c} & -{\epsilon }\end{array})$$

here $\epsilon$ and *T*_*c*_ are the potential difference and tunneling between the two dots, respectively. Using dynamical nuclear spin polarizations^45^ or an integrated micromagnet^46,47^ can produce the magnetic field gradient term Δ*B*.

A superconducting transmission line resonator is modeled as a circuit of length *L* with capacitance per unit length *C*_*R*_ and impedance *Z*_*R*_. The voltage of the resonator is quantized as^47^: $\hat{V}={\sum }_{k}\,\sqrt{\frac{{\omega }_{k}}{L{C}_{R}}({\hat{a}}_{k}+{\hat{a}}_{k}^{\dagger })}$, where *â* and ${\hat{a}}^{\dagger }$ are the annihilation and creation operators of the resonator modes, respectively. In practice, we usually focus on the case when the energy splitting between eigenstates of Eq. (1) is comparable to the frequency of the fundamental mode of the resonator. Thus, ignoring the other higher energy modes, the resonator can be described by the standard Hamiltonian:

2 $${H}_{R}={\omega }_{r}({\hat{a}}^{\dagger }\hat{a}+\frac{1}{2})$$

where the resonator is in the lowest energy mode with the characterized frequency ${\omega }_{r}=\frac{k}{{C}_{R}{Z}_{R}}$ and the corresponding wave vector $k=\frac{\pi }{L}$.

As shown in Fig. 1a, one of the dots is capacitive coupled to the resonator. The interaction between the quantum dot and the resonator consists of two contributions ${\epsilon }+e\hat{V}\frac{{C}_{c}}{{C}_{QD}}$^48^: the gate voltage $\epsilon$ of the double dot, and the voltage $\hat{V}$ of the resonator. Here *C*_*QD*_ is the capacitance of the double dot, *C*_*c*_ is the capacitance between the dot and the resonator. The interaction Hamiltonian can be described as:

3 $${H}_{I}=g(\hat{a}+{\hat{a}}^{\dagger })|(0,2)S\rangle \langle (0,2)S|,$$

where the quantity $g=e\frac{{C}_{c}}{{C}_{QD}L{C}_{R}}\sqrt{\frac{\pi }{{Z}_{R}}}$ is the “bare” coupling between the double dot and the resonator.

### Tunable coupling between the electron spin qubit and the resonator

We consider two different working regimes of the qubit. When ${\epsilon }\ll 0$, using adiabatic elimination method^44^, we can deduce the effective Hamiltonian of the qubit from Eq. (1):

4 $${H}_{q}=(\begin{array}{cc}0 & {\rm{\Delta }}B\\ {\rm{\Delta }}B & -J\end{array})$$

with the exchange coupling $J({\epsilon })=\frac{{\epsilon }}{2}+\sqrt{\frac{{{\epsilon }}^{2}}{4}+{T}_{c}^{2}}$. Based on this Hamiltonian, there are various experiments to implement single-qubit universal gates and two-qubit controlled-phase gate for such singlet-triplet qubit^22,30,45^. Therefore we refer it as “control regime” in the following.

When ${\epsilon } \sim 0$, it is convenient to consider the Hamiltonian in the subspace of singlet states $|(1,1)S\rangle$ and $|(0,2)S\rangle$:

5 $${H}_{s}=(\begin{array}{cc}0 & {T}_{c}\\ {T}_{c} & -{\epsilon }\end{array}).$$

In the eigenbasis |*e*〉 and |*g*〉 of the Hamiltonian *H*_*s*_, we obtain the total Hamiltonian (the derivation is provided in the Supplementary Information):

6 $${H}_{t}={H}_{s}+{H}_{R}+{H}_{I}=\frac{{\omega }_{s}}{2}{\hat{\sigma }}_{z}+{\omega }_{r}({\hat{a}}^{\dagger }\hat{a}+\frac{1}{2})-{g}_{x}(\hat{a}+{\hat{a}}^{\dagger }){\hat{\sigma }}_{x}+{g}_{z}(\hat{a}+{\hat{a}}^{\dagger }){\hat{\sigma }}_{z},$$

where ${\hat{\sigma }}_{i}$ are the Pauli matrices, energy level splitting ${\omega }_{s}=\sqrt{{{\epsilon }}^{2}+4{T}_{c}^{2}}$, the coupling coefficients ${g}_{x}=\frac{1}{2}g\,\sin \,2\theta$, ${g}_{z}=\frac{1}{2}g\,\cos \,2\theta$, and the mixing angle $\theta =\frac{1}{2}arctan(\frac{2{T}_{c}}{{\epsilon }})$.

The Hamiltonian of Eq. (6) has several remarkable features of the hybrid system. Firstly, the coupling between the double dot and the resonator arises in both the longitudinal and transverse directions. The transverse coupling allows exchange between spin qubit and resonator, while the existence of a longitudinal coupling can play a role for designing specific protocols. Secondly, the coupling strength depends on the mixing angle *θ*, which is directly proportional to the gate voltage $\epsilon$. As shown in Fig. 1b, the spin-photon coupling can be tuned by the gate voltage $\epsilon$ and inter-dot tunneling *T*_*c*_, which provides possibility to seek a set of controllable parameters for various quantum information tasks. For example, as detailed in the following, we can find the working point around $\epsilon$ = 0 which maximizes the coupling strength and minimizes the noise effects for the measurement protocol. Correspondingly, we refer it as “measurement regime” for the present architecture.

### Evolution equations of the hybrid system

The resonator can be used for readout of the qubit state. This can be realized by microwave irradiation of the resonator and then detecting the transmitted photons. A microwave drive of frequency *ω*_*m*_ on the resonator can be modeled by^48^

7 $${H}_{d}={V}_{m}({\hat{a}}^{\dagger }{e}^{-i{\omega }_{m}t}+\hat{a}{e}^{i{\omega }_{m}t}).$$

The dynamics of the whole system in presence of dissipation and dephasing is described by a master equation:

8 $$\rho =-i[{H}_{t}+{H}_{d},\rho ]+\kappa {\mathscr{D}}[\hat{a}]\rho +\Gamma {\mathscr{D}}[{\hat{\sigma }}_{-}]\rho $$

where *ρ* is the density matrix of the coupled system, ${\mathscr{D}}[\hat{A}]\rho =\hat{A}\rho {\hat{A}}^{\dagger }-{\hat{A}}^{\dagger }\hat{A}\rho /2-\rho {\hat{A}}^{\dagger }\hat{A}/2$ is the Lindbald-type operator^49,50^.

In order to gain further information about the dynamics, we derive the equations of motions for the whole system. The relevant quantities are the expectation value of the qubit operators 〈${\hat{\sigma }}_{i}$〉 and the resonator field operator 〈$\hat{a}$〉. We follow a “semi-classical” procedure which simply factorizes all higher-order moments^50^. It is often a good place to start as it captures the underlying dynamical properties of the problem. We can obtain the following equations of motions of the whole system in the rotating frame:

9 $$\frac{d\langle \hat{a}\rangle }{dt}=-\,i{\Delta }_{rm}\langle \hat{a}\rangle +i{g}_{x}\langle {\hat{\sigma }}_{-}\rangle -i{g}_{z}{e}^{i{\omega }_{m}t}\langle {\hat{\sigma }}_{z}\rangle -i{V}_{m}-\frac{\kappa }{2}\langle \hat{a}\rangle $$

10 $$\frac{d\langle {\hat{\sigma }}_{-}\rangle }{dt}=-\,i{\Delta }_{sm}\langle {\hat{\sigma }}_{-}\rangle -i{g}_{x}\langle \hat{a}\rangle \langle {\hat{\sigma }}_{z}\rangle -2i{g}_{z}({e}^{-i{\omega }_{m}t}\langle \hat{a}\rangle +{e}^{i{\omega }_{m}t}{\langle \hat{a}\rangle }^{\dagger })\langle {\hat{\sigma }}_{-}\rangle -\frac{{\Gamma }_{1}}{2}\langle {\hat{\sigma }}_{-}\rangle $$

11 $$\frac{d\langle {\hat{\sigma }}_{z}\rangle }{dt}=2i{g}_{x}(\langle \hat{a}\rangle \langle {\hat{\sigma }}_{+}\rangle -\langle {\hat{a}}^{\dagger }\rangle \langle {\hat{\sigma }}_{-}\rangle )-{\Gamma }_{1}(\langle {\hat{\sigma }}_{z}\rangle +1)$$

here *Δ*_*rm*_ = *ω*_*r*_ − *ω*_*m*_ and *Δ*_*sm*_ = *ω*_*s*_ − *ω*_*m*_ are the detuning of the measurement microwave fields from the resonator and qubit frequency, respectively. These equations have some advantages compared to the master Eq. (8): each term in the equations has apparent contribution from the Hamiltonian. In addition, they are much faster to solve numerically and even can be solved analytically in particular cases. Actually, we have checked that in the situations discussed in this paper, these equations have given the same results as those from directly solving the master Eq. (8).

### Decoherence effects in the hybrid system

In the model, we include three different decoherence effects:

The first decoherence source is the photon dissipation of the resonator. Here we model it via the $\kappa {\mathscr{D}}[\hat{a}]\rho$ term in the master equation. We introduce the decay rate as *κ* = *ω*_*r*_/*Q*, where *Q* is the quality factor of the resonator. The decay rate *κ* is of the order 0.1 MHz for the experimental reported number *Q* ≈ 1 × 10^4^ ^36^.

The second decoherence source is the relaxation of the qubit system in a time *T*_1_. The relaxation mechanism is the coupling between the charge degree of freedom of the qubit and a phonon bath^44^. Here we model it via the ${\Gamma }_{1}{\mathscr{D}}[{\hat{\sigma }}_{-}]\rho$ term in the master equation. We note that there is also the cavity-induced relaxation of the qubit, the Purcell effect. However, the Purcell effect can be suppressed by increasing the detuning between the qubit and the resonator.

The third decoherence channel arises from two sources of noise in GaAs quantum dot systems: One is the charge noise due to the fluctuations of the charge impurities or control pulse imperfections^21,51,52^; the other is the spin noise due to the fluctuations of the nuclear spin bath^53,54^. We model these two noises source as random values ${\delta }_{\epsilon }$ and *δΔB* in the Hamiltonian, i.e., as perturbations around the gate voltage $\epsilon$ and the magnetic-field gradient *ΔB* terms in the Hamiltonian. Because gate operation times are much faster, the charge noise ${\delta }_{\epsilon }$ and the spin noise *δΔB* can be treated as the low-frequency fluctuations with a Gaussian distribution.

We numerically solve the Eq. (8) to simulate the dynamics of the hybrid system. At each run, we choose the value of the charge noise term ${\delta }_{\epsilon }$ and the spin noise term *δ*Δ*B* from a Gaussian distribution. Then the results are averaged for many realizations of the random noises. We note that this average method has been applied in a variety of quantum dot systems^55,56^.

### Resonator response for the spin singlet-triplet qubit operations

The dynamics of the spin qubit can be represented by applying the unitary operation matrices ${\hat{R}}_{x}$ and ${\hat{R}}_{z}$ which give rise to a rotation on the Bloch sphere around the *x* axis and around the *z* axis, respectively. Here we exemplify the readout protocol for the specific case of ${\hat{R}}_{x}$ gate operation, which address the important issues about how to probe the gate operations of spin qubit in the present architecture.

**Figure 2:**
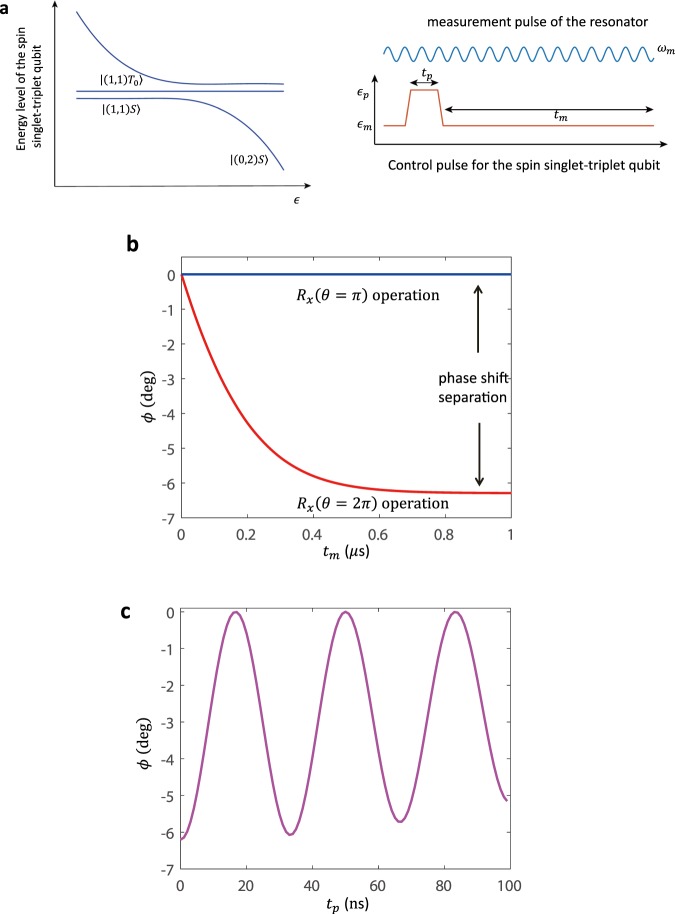
(**a**) (left) The energy level diagram of the spin single-triplet qubit in double quantum dot. (right) Schematic showing the sequence for coherent control of the spin qubit and measurement of the resonator signal. (**b**) The phase of resonator field *ϕ* as a function of the measurement time *t*_*m*_. The signal shows the resonator response dynamics, conditioned on the spin qubit state. (**c**) The phase of resonator field *ϕ* as a function of the gate operation time *t*_*p*_, showing coherent oscillations of spin qubit states.

Figure 2a shows the pulse scheme used for the qubit gate operation and resonator measurement protocol. Applying the pulse, we manipulate the qubit in the control regime and then move to the measurement regime for the readout of qubit state. The spin qubit starts from the initial point in the $|(0,2)S\rangle$ state. We apply a rapid adiabatic passage (RAP) which is fast with respect to the gradient energy Δ*B* but adiabatic with respect to *T*_*c*_. Therefore the spin qubit adiabatically evolves into the $|(1,1)S\rangle$ state. Waiting at the point where *J* = 0 for a time *t*_*p*_, a *X* rotation ${\hat{R}}_{x}$ is realized^44^. For example, a ${\hat{R}}_{x}$(*θ*) rotation with *θ* = *π* will drive a spin transition from $|(1,1)S\rangle$ to $|(1,1){T}_{0}\rangle$. The reversed pulse back to ${{\epsilon }}_{m}$ adiabatically transfers the state $|(1,1)S\rangle$ to $|(0,{\rm{2}})S\rangle$, which is coupled to the resonator; while the state $|(1,1){T}_{0}\rangle$ is unchanged, which still has one electron in each dot.

Simultaneously, the resonator is continuously driven at the amplitude of *V*_*m*_ and frequency of *ω*_*m*_. The resulting spin state is probed by the resonator transmission field for a measurement time *t*_*m*_. The resonator field is sensitive to spin qubit dynamics owing to the different qubit-resonator coupling of spin state $|(0,{\rm{2}})S\rangle$ and $|(1,1){T}_{0}\rangle$, thereby allowing for measurement of the spin qubit states.

When a spin qubit operation ${\hat{R}}_{x}$ is performed, we can detect both the phase *ϕ* and the amplitude of the coherent microwave field transmitted through the resonator. The temporal component *ϕ* at the output of the resonator is related to the dynamics of the resonator-qubit system by *ϕ*(*t*) = *arg*{*i*〈$\hat{a}$〉}, where $\hat{a}$ is the resonator field. In Fig. 2b, we plot the phase shift *ϕ* as a function of measurement time *t*_*m*_. The time dependence of the resonator signal is numerically solved by the equations of motions (9–11) with a set of typical realistic parameters^36^. On one hand, here we use the resonator frequency as *ω*_*r*_ = 2*π* × 6.4 *GHz*, with a photon decay rate of *κ* = 2*π* × 1.7 *MHz*. The resonator is continuously driven at the amplitude of ${V}_{m}=\sqrt{\kappa /2}$ which populates the resonator with few photons on average, and the working frequency *ω*_*m*_ = *ω*_*r*_. On the other hand, the spin qubit bias $\epsilon$ can be electrically adjusted by the external gate voltage, and we set the working point ${{\epsilon }}_{m}=0$ for measurement protocol in the following. The tunneling between the dots is *T*_*c*_ = 10 *μeV*, and a magnetic field gradient energy is Δ*B* = 0.06 *μeV*. The qubit decay rate is *Γ*_1_ = 24 *MHz*. In practice, the environment noises have significant influence on the spin-qubit dynamics. The charge noise satisfies a Gaussian distribution with the standard deviation ${\sigma }_{\in }\approx 4\,\mu eV$^52^; while the spin noise satisfies a Gaussian distribution with the standard deviation *σ*_Δ*B*_ ≈ 0.5 *mT*^53^. The qubit-resonator coupling *g* = 2*π* × 30 *MHz* and the effective component *g*_*x*_ and *g*_*z*_ can also be adjusted using the external gate voltage.

There are several features in the dynamics of qubit-resonator system as plotted in Fig. 2b,c. First, in Fig. 2b, we study the resonator response to the ${\hat{R}}_{x}$(*θ*) rotation with *θ* = *π* or *θ* = 2*π* for a full characterization of the gate operation. As expected, no phase shift is observable for the *θ* = *π* pulse since the spin qubit is prepared in the state $|(1,1){T}_{0}\rangle$. The response to the *θ* = 2*π* pulse is distinguishable from the one to the *θ* = 2*π* pulse since the spin qubit is prepared in the state $|(1,1)S\rangle$. Second, applying a control pulse to manipulate the spin qubit, we can obtain a clear periodical coherent oscillation pattern in the phase shift of the resonator as shown in Fig. 2c. This demonstrates that the resonator phase plays a role as an effective probe for the qubit operations. Third, at the start of the measurement protocol the phase shift has a value *ϕ* = 0 corresponding to the initial state of the resonator. When the spin qubit is prepared in the state $|(1,1)S\rangle$ induced by control pulse, the resonator phase *ϕ* rises rapidly towards to a maximum value *ϕ*_*max*_ = −6.2 *deg*. The response time scale of *ϕ* is about *T*_*r*_ = 1/*κ* ≈ 100 *ns*, i.e., the photon life time of the resonator. We can define the measurement signal separation *C* = −*ϕ*_*max*_ as a figure of merit to characterize the performance of the measurement protocol (the discussion about the signal-to-noise ratio in the measurement process is provided in the Supplementary Information).

**Figure 3:**
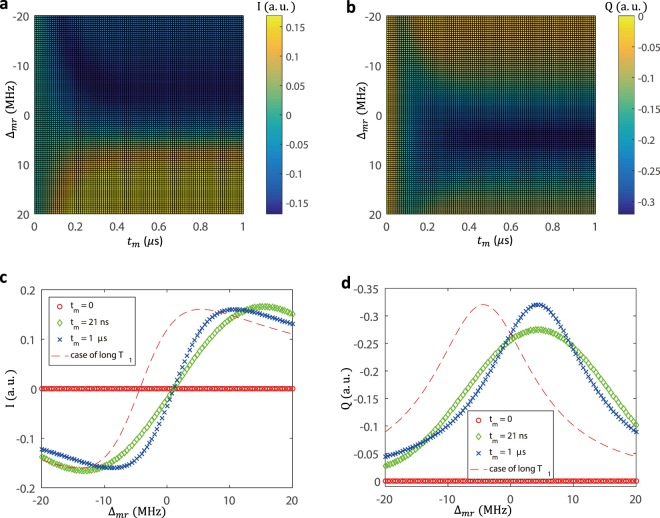
(**a**) and (**b**) The quadratures of the resonator fields *I* and *Q* are plotted as a function of the measurement frequency detuning Δ_*mr*_ = *ω*_*m*_ − *ω*_*r*_ and the measurement time *t*_*m*_. (**c**) and (**d**) To interpret the time and frequency dependence of the resonator signal, it is instructive to plot *I* and *Q* as a function of Δ_*mr*_ at different fixed times *t*_*m*_.

To gain more information about the measurement protocol, it is instructive to plot the response signal as a function of the measurement time *t*_*m*_ and working frequency *ω*_*m*_, as shown in Fig. 3a,b. These quadrature components of the resonator field are related to equations of motions of the system by^48^:

12 $$I(t)=\mathrm{Re}\langle \hat{a}(t)\rangle ,Q(t)=Im\langle \hat{a}(t)\rangle $$

For clarity, the quadratures are plotted in Fig. 3c,d as a function of *ω*_*m*_ at fixed times *t*_*m*_. When applying a ${\hat{R}}_{x}$(*θ* = 2*π*) control pulse, the spin qubit is prepared in the state $|(1,1)S\rangle$. In Fig. 3(c), the characteristic frequency of the resonator signal shifts, but the signal responds only on a time scale corresponding to the photon lifetime *T*_*r*_ = 1/*κ*. At time *t*_*m*_ = 21 *ns*, the shift of the resonator signal curve toward higher frequency is clearly visible. At *t*_*m*_ = 2 *μs*, the line shape of the resonator spectrum is centered at maximum frequency. For comparison, we plot the signal for an ideal case where the qubit lifetime is much longer than the photon lifetime *T*_1_ ≫ *T*_*r*_. The line shape of the resonator spectrum is centered at lower frequency at this limit. Thus the interplay of the resonator rise time *T*_*r*_ and the qubit decay time *T*_1_ determines the dynamics of the resonator transmission signal. The same considerations can explain the features observed in the response trace in Fig. 3d.

### Optimal working points of the resonator signal

For many quantum information applications, it is highly desirable to tune qubit-resonator coupling on for quantum-state transfer or off for qubit-state manipulation. Many CQED systems have almost constant coupling, or the coupling strength can only adjust by a few percent. Here the coupling strength *g*_*x*_ and *g*_*z*_ can be electrically controlled by modifying $\epsilon$ and *T*_*c*_ through the external gate voltage. An important property of the proposed spin qubit-resonator system is the existence of a longitudinal coupling. The strength of the transverse coupling can reach several tens of MHz around ${{\epsilon }}_{m}$ = 0, while the longitudinal coupling can switch on at the same order as detuned from ${{\epsilon }}_{m}$ = 0. These observation can be understood by considering the $\epsilon$ dependence of the charge states of the spin singlet-triplet qubit. Around $\epsilon$ = 0, the two electrons are localized in the right dot and forms |(0, 2)〉 charge state. In this regime, the displacement of the electron wavefunction leads to a large electric dipole moment. As a result, the spin qubit experiences a large qubit-resonator coupling *g* in the electric field of the resonator. In contrast, with ${\epsilon }\ll 0$ the two electrons are delocalized across the double quantum dot and works with the delocalized electronic wavefunctions |(1, 1)*S*〉 and |(1, 1)*T*_0_〉, where the number correspond to the electron being in the left and right dot, respectively. In this regime, the displacement of the electron wavefunction is expected to be negligible, substantially suppressing the qubit-resonator coupling mechanism. The large difference in the electric dipole moment between the different charge configuration regimes results in the effective spin qubit-resonator coupling at working point ${{\epsilon }}_{m}$ = 0 of approximately three times larger compared to working point ${{\epsilon }}_{m}$ = 6 *μeV*.

**Figure 4:**
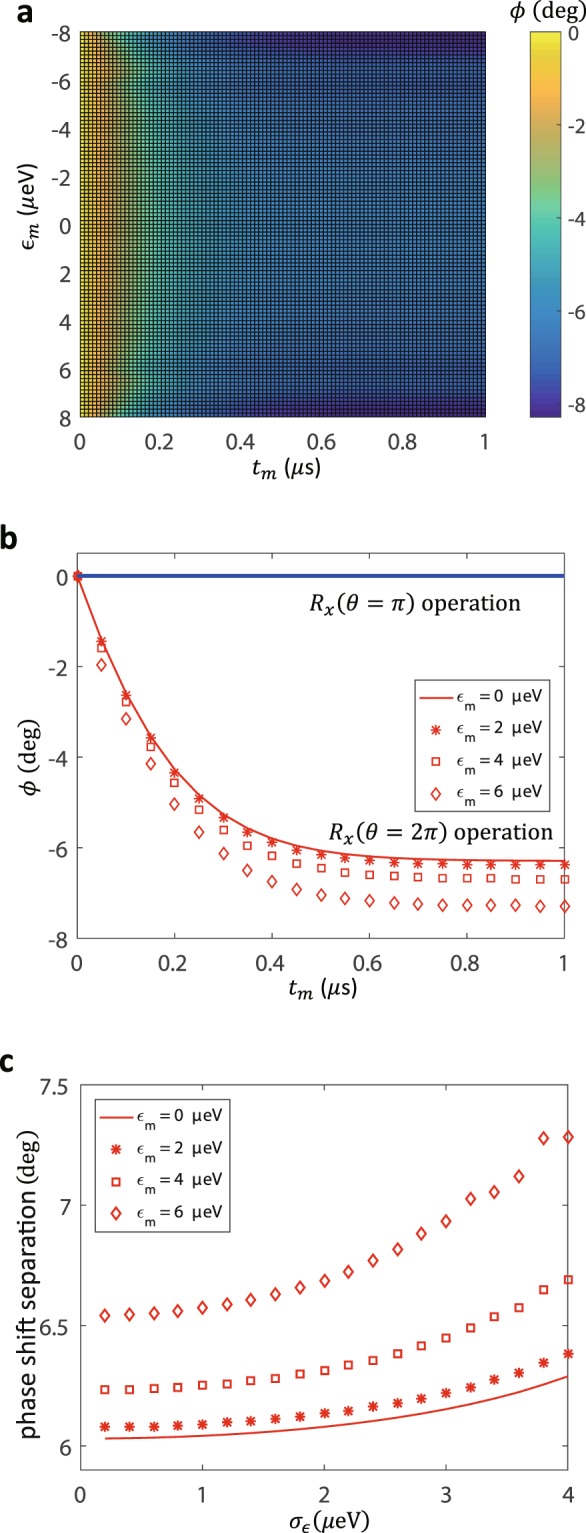
(**a**) The phase of resonator field *ϕ* as a function of working point ${{\epsilon }}_{m}$ and measurement time *t*_*m*_. (**b**) For clarity, the phase signal *ϕ* as a function of measurement time *t*_*m*_ are plotted at working point ${{\epsilon }}_{m}$ = 0, 2, 4, 6 *μeV*, respectively. (**c**) For comparison, the measurement signal separation is shown as a function of the standard deviation of charge noise ${\sigma }_{\epsilon }$ at different working points.

It is interesting to consider the measurement protocol at different working points ${{\epsilon }}_{m}$ to explore the role of different couplings. In Fig. 4a,b we show the resonator signal *ϕ* as a function of measurement time *t*_*m*_ and working point ${{\epsilon }}_{m}$. At ${{\epsilon }}_{m}$ = 0, the maximum phase shift *ϕ*_*max*_ = −6.2 *deg* is observed (with a transverse coupling of *g*_*x*_ = 188 *MHz*) that is somewhat smaller than the value of *ϕ*_*max*_ = −7.2 *deg* obtained at ${{\epsilon }}_{m}$ = 6 *μeV* (with a transverse coupling of *g*_*x*_ = 57 *MHz* and a longitudinal coupling of *g*_*z*_ = 74 *MHz*).

Moreover, we analyze the effect of charge noise, rather than conventional magnetic noise, which is the relevant decoherence mechanism as well as the experimental imperfection for spin qubit system^21,52,57–60^. We compare the measurement signal separation as a function of the charge noise at ${{\epsilon }}_{m}$ = 0, 2, 4, 6 *μeV*. We can find a substantially smaller shift of the measurement signal separation at ${{\epsilon }}_{m}$ = 0 *μeV* than that at ${{\epsilon }}_{m}$ = 6 *μeV*, suggesting that the measurement performance is robust against the environment noise. We seek ${{\epsilon }}_{m}$ = 0 *μeV* as the optimal working point for two reasons. First, at this point the energy level splitting ${\omega }_{s}=\sqrt{{{\epsilon }}^{2}+4{T}_{c}^{2}}$ is insensitive to first-order changes in the term $\epsilon$. Therefore this suppresses the fluctuations ${\delta }_{\epsilon }$ due to the environment charge noise or control electronics noise, which are found to be the dominant dephasing sources in spin-qubit implementations. Second, the longitudinal coupling *g*_*z*_ vanishes at the point ${{\epsilon }}_{m}$ = 0, in which case the transverse coupling *g*_*x*_ is maximized. Alternatively, the effective coupling between spin-qubit and resonator is maximized at the point where dephasing effects are minimized.

## Discussion

In summary, we comprehensively analyzed the scenario of the measurement protocol in a spin singlet-triplet qubit-resonator coupled system. We analyze a set of quantities of the resonator signal that allows us to make clear picture about the system parameters needed for a faithful experimental implementation, where the parameter space is spanned by the measurement time, detecting frequency, and working points. When looking at the time trace of the phase shift of the resonator, the characteristic dynamics indicate that the phase shift of the resonator field can probe the gate operations of the spin singlet-triplet qubit. For the small time, the resonator rise dynamics dominates and produces the signal. Moreover, at the working point ${{\epsilon }}_{m}$ = 0, a qubit-resonator coupling of several tens of MHz can be reached, allowing for a strong measurement signal separation and robust against the charge noise. Our work paves a way to further applications using the resonator as the highly efficient probe for spin-based quantum information tasks. We highlight possible directions of research going beyond our present work. (i) While we use spin singlet-triplet qubit state as the main example of our technique, our theoretical approach generalizes immediately to other spin encodings. (ii) In this work we focus on the simple measurement protocol of the resonator field. With further sophisticated protocols, these basic ingredients could be readily implemented, giving rise to single-shot readout, quantum nondemolition measurement, and parity measurement for quantum error correction.

## Electronic supplementary material

Supplementary Information
