# Supplementary material for: Dynamics of probing a quantum-dot spin qubit with superconducting resonator photons
Source: Sci Rep. 2018 Oct 25;8:15761. doi: 10.1038/s41598-018-34108-0 (PMC6202405; doi:10.1038/s41598-018-34108-0)
Supplement: Supplementary file 1 — Supplementary Information [file 41598_2018_34108_MOESM1_ESM.pdf]

Dynamics of probing a quantum-dot spin qubit with superconducting resonator photons : Supplemental information

X. Y. Zhu, T. Tu, A. L. Guo, Z. Q. Zhou, G. C. Guo, and C. F. Li
(Dated: August 23, 2018)

EFFECTIVE HAMILTONIAN FOR THE COMBINED SYSTEM

We consider a double quantum dot structure near the charge degeneracy point $\epsilon = 0$. An effective Hamiltonian for the double dot system can described as

$$H_s = \frac{\epsilon}{2}\widetilde{\sigma}_z + T_c\widetilde{\sigma}_x. \tag{S1}$$

Here $\widetilde{\sigma}_z$ and $\widetilde{\sigma}_x$ are the Pauli matrix defined in the subsapce of $|(1,1)S\rangle$ and $|(0,2)S\rangle$, ϵ and T_c are the detuning and tunneling betwen the two dots, respectively.

The superconducting cavity is usually given as a resonator with the characterized frequency ω_r:

$$H_r = \omega_r(a^+a + \frac{1}{2}). \tag{S2}$$

Here a^+ and a are the creation and annihilation operators. The electric voltage induced by the resonator field is quantized as

$$\widehat{V} = \sqrt{\frac{\omega_r}{LC_R}}(a + a^+), \tag{S3}$$

with the length L and capacitance C_R of the resonator. When the double quantum dot is fabricated inside the superconducting cavity, the interaction between the double dot and the resonator can be given as [1]:

$$H_I = \frac{C_c}{C_{QD}}e\widehat{V}\widetilde{\sigma}_z. \tag{S4}$$

Here C_c is the capacitive coupling between the dot and the resonator, and C_{QD} is the capacitance of the dot system. Introducing the coupling coefficient

$$g = e\frac{C_c}{C_{QD}}\sqrt{\frac{\omega_r}{LC_R}}, \tag{S5}$$

we can rewire the interaction Hamiltonian as

$$H_I = g(a + a^+)\widetilde{\sigma}_z. \tag{S6}$$

Taking into account all the terms of the combined system, we obatin

$$H_{eff} = H_s + H_r + H_I = \frac{\epsilon}{2}\widetilde{\sigma}_z + T_c\widetilde{\sigma}_x + \omega_r(a^+a + \frac{1}{2}) + g(a + a^+)\widetilde{\sigma}_z. \tag{S7}$$

For the quantum dot system, the Hamiltonian in the basis of $|(1,1)S\rangle$ and $|(0,2)S\rangle$ is

$$H_s = \begin{pmatrix} \frac{\epsilon}{2} & T_c \\ T_c & -\frac{\epsilon}{2} \end{pmatrix}. \tag{S8}$$

Diagonalizating the above matrix, there are two eigen states

$$|g\rangle = \begin{pmatrix} \cos\theta \\ \sin\theta \end{pmatrix}, \tag{S9}$$

and

$$|e\rangle = \begin{pmatrix} -\sin\theta \\ \cos\theta \end{pmatrix}. \tag{S10}$$

with eigen energies

$$E_g = -\sqrt{\frac{\epsilon^2}{4} + T_c^2}. \tag{S11}$$

and

$$E_e = \sqrt{\frac{\epsilon^2}{4} + T_c^2}. \tag{S12}$$

Here we simplify the algerba by letting the mixing angle θ as

$$\sin 2\theta = -\frac{T_c}{\sqrt{\frac{\epsilon^2}{4} + T_c^2}}, \tag{S13}$$

and

$$\cos 2\theta = \frac{\frac{\epsilon}{2}}{\sqrt{\frac{\epsilon^2}{4} + T_c^2}}. \tag{S14}$$

Working in the eigen basis of $|e\rangle$ and $|g\rangle$, we can transform the Hamiltonian (S7) into

$$H_{eff} = \frac{\omega_s}{2}\sigma_z + \omega_r(a^+a + \frac{1}{2}) - g_x(a + a^+)\sigma_x + g_z(a + a^+)\sigma_z. \tag{S15}$$

Here the energy splitting $\omega_s = \sqrt{\epsilon^2 + 4T_c^2}$, σ_z and σ_x are the Pauli matrix in the basis of $|e\rangle$ and $|g\rangle$, the transverse and longitudinal coupling strength are defined as $g_x = \frac{1}{2}g\sin 2\theta$, $g_z = \frac{1}{2}g\cos 2\theta$, respectively. These results are the Eq. (11) in the main text.

SIGNAL TO NOISE IN THE MEASUREMENT PROCESS

For quantum information processing, a fast and high fidelity readout of qubit states plays an essential role. The readout strategy prsented in this paper depends on the setup in which the electron spin qubit in quantum dots is coupled to microwave resonstor. With the interaction between the qubit and resonator, the resonator field is displaced in a qubit state-dependent fashion. For the qubit is in state $|0\rangle = |(1,1)T_0\rangle$ and $|1\rangle = |(1,1)S\rangle$, the phase shift of the resonator field corresponds to $\phi_{|0\rangle}$ and $\phi_{|1\rangle}$. Thus the signal of the resonator field contains the complete information of the qubit states.

The measurement can be characterized by its signal-to-noise (SNR). The magnitude of the phase shift is proportional to the number of photons in the resonator

$$n_{sig} = \overline{n}\kappa t_m/2. \tag{S16}$$

Here $\overline{n}$ is the mean number of photons in the resonator, κ is the rate of photons leaving the resonator, t_m is the integration time during which the signal accumulates. A conservaticve estimate of the noise of the resonator field is given as

$$\delta n = k_B T_n/\hbar\omega_r. \tag{S17}$$

Here T_n is the noise temperature of the readout circuit, k_B and $\hbar$ are the Boltzmann constant and Planck constant. Since the intergation time is set by the decay time of the qubit $T_1 = 1/\Gamma_1$, we obtain the formula

$$\mathrm{SNR} = \frac{\overline{n}}{\delta n}\frac{\kappa}{2\Gamma_1}. \tag{S18}$$

In practice, the detector operates in the low temperature mK regime for providing low noise, while the signal can be made larger by driving the resonator to a higher number of photons $\overline{n} = n_{crit} \approx 100\delta n$ [1]. For the realistic parameters of $\Gamma_1 = 24$ MHz and $\kappa = 10$ MHz in typical experiments, the value of SNR is calculated about 21 in our measurement scheme. To further improve the readout SNR, the quantum amplifiers are developed to increase the magnitude of the signal and reach the quantum noise limit [2–4].

[1] Blais, A. Huang, R. S., Wallraff, A. Girvin, S. M. & Schoelkopf, R. J. Cavity quantum electrodynamics for superconducting electrical circuits: An architecture for quantum computation. *Phys. Rev. A* **69,** 062320 (2004).

[2] Bergeal, N., et al. Phase-preserving amplification near the quantum limit with a Josephson ring modulator. *Nature* **465**, 64 (2010).

[3] Macklin, C., et al. A near-quantum-limited Josephson traveling-wave parametric amplifier, *Science* **350**, 307 (2015).

[4] Krantz, P., et al. Single-shot read-out of a superconducting qubit using a Josephson parametric oscillator, *Nat. Commun.* **7**, 11417 (2016).
